# Supplementary material for: The causal relationship between the human gut microbiota and pyogenic arthritis: a Mendelian randomization study
Source: Front Cell Infect Microbiol. 2024 Nov 26;14:1452480. doi: 10.3389/fcimb.2024.1452480 (PMC11629706; doi:10.3389/fcimb.2024.1452480)
Supplement: Supplementary file 1 [file DataSheet1.zip › mendelian test/ebi-a-GCST90027544.csv_forest.pdf]

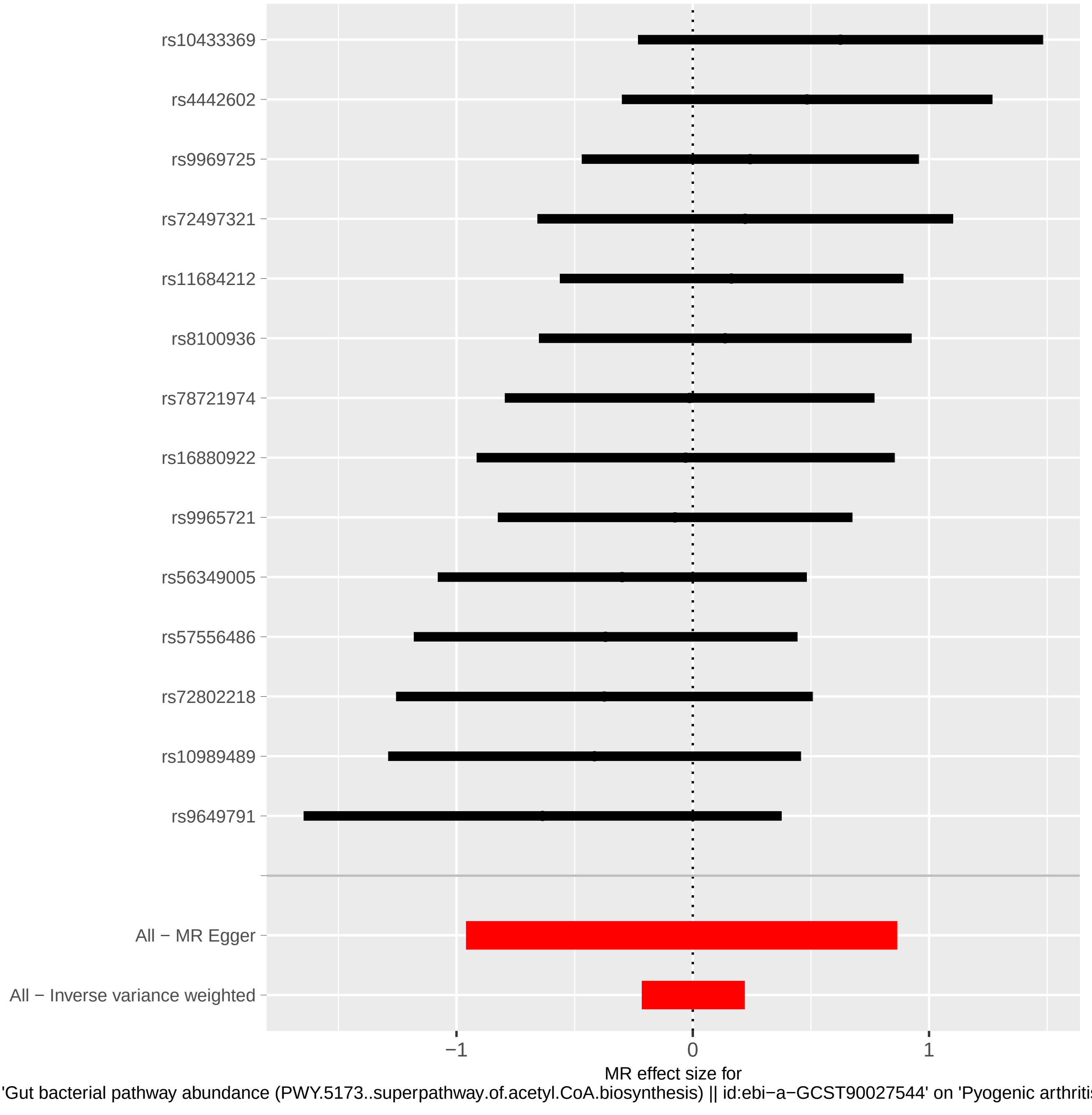
rs10433369
rs4442602
rs9969725
rs72497321
rs11684212
rs8100936
rs78721974
rs16880922
rs9965721
rs56349005
rs57556486
rs72802218
rs10989489
rs9649791
All − MR Egger
All − Inverse variance weighted
−1
0
1
MR effect size for
'Gut bacterial pathway abundance (PWY.5173..superpathway.of.acetyl.CoA.biosynthesis) || id:ebi−a−GCST90027544' on 'Pyogenic arthritis
